# *Lentinula edodes* Stalk Polysaccharide Coating Extends the Shelf-Life of *Agaricus bisporus* by Modulating Respiration and Energy Metabolism

**DOI:** 10.3390/foods14244172

**Published:** 2025-12-05

**Authors:** Wenjuan Yang, Tingting Xia, Ruifeng Miao, Hui Long, Jing Wang, Nan Li, Yanni Zhao, Fuxin Chen, Yuxi Guo, Pin Gong

**Affiliations:** 1 School of Food Science and Engineering, Shaanxi University of Science and Technology, Xi’an 710021, China; yangwenjuan@sust.edu.cn (W.Y.); xiatingting@sust.edu.cn (T.X.);; 2 Key Laboratory of Precision Nutrition and Functional Product Development in Xi’an, Shaanxi University of Science and Technology, Xi’an 710021, China; 3 School of Chemistry and Chemical Engineering, Xi’an University of Science and Technology, Xi’an 710054, China

**Keywords:** browning and softening, exogenous polysaccharides, mechanism

## Abstract

This study comprehensively evaluated the effect of a Shiitake Stalk Isolated Polysaccharide (LEP) coating on *Agaricus bisporus*. The study findings revealed that the application of 1.5% LEP reduced weight loss, maintained firmness and chewiness, as well as the nutritional indices of mushrooms, extended the shelf life by 9 days, and maintained freshness for up to 15 days compared to the control. In addition, LEP enhanced the activities of various ROS metabolism-related enzymes such as superoxide dismutase (SOD), catalase (CAT), and peroxidase (POD) and significantly reduced the accumulation of hydroxides. LEP treatment during storage maintained high antioxidant activity. Additionally, LEP treatment is associated with increased activity of CCO, SDH, H^+^-ATPase, and Ca^2+^/ATPase. LEP also preserved mitochondrial membrane structural integrity, which helps to prolong shelf life. The results suggest that the LEP’s polysaccharide component may serve as a beneficial additive to prevent the deterioration of mushrooms during preservation and provide an opportunity to expand the use of LEP in edible mushroom preservation.

## 1. Introduction

*Agaricus bisporus* is one of the most commonly cultivated edible mushroom species worldwide [1]. China is one of the leading producers of *Agaricus bisporus*. As of 2022, the annual production of *A. bisporus* in China reached 1,572,500 tonnes. The main production areas in China are located in the regions of Jiangsu, Henan, Fujian, and Guangxi, which together account for about 70% of national production [2]. The quality of mushrooms is determined by color, texture, cleanliness, and flavor, with color being the first attribute perceived and evaluated by consumers. This is particularly important for the consumer acceptance of *A. bisporus* [1,3].

Postharvest mushrooms continuously respire and require a proper energy supply to sustain their life activities, which is essential for maintaining freshness [3,4,5]. Numerous studies have shown that postharvest senescence, quality deterioration, browning, and softening of mushrooms are associated with cellular function and the level of cellular energy required for survival. For example, *Hypsizygus marmoreus* showed significant reductions in succinate dehydrogenase, cytochrome oxidase, H^+^-ATPase, and Ca^2+^-ATPase, as well as significant reductions in energy homeostasis, during postharvest storage and environmental stresses such as cold damage [6,7].

Therefore, preservation methods based on the regulation of respiration and energy metabolism have attracted increasing attention. The addition of exogenous substances is a typical strategy. Guo et al. [6] explored the effects of Benzothiadiazole (BTH) coating treatment on apple fruit development and mitochondrial energy metabolism. BTH treatment significantly reduced spot diameter in *Penicillium expansum*-infested apples. Furthermore, BTH treatment improved the activity of mitochondrial respiratory metabolism-related enzymes, as well as high ATP levels under all storage conditions [6]. EBR treatment promoted the activity and transcription of enzymes related to energy and sucrose metabolism, maintained higher ATP content and energy status, and upregulated the transcript levels of energy metabolism-related genes, including *PpCCO*, *PpSDH*, and *PpH^+^-ATPase* [6]. Physical preservation methods based on respiration and energy metabolism are another common strategy. Light irradiation resulted in a decrease in adenosine triphosphate, adenosine diphosphate, adenosine monophosphate, and energy charge. This was due to a decrease in the activity of energy-metabolizing enzymes. The application of nanocomposites delayed the disruption of mitochondrial microstructure and the decrease in ATPase activity, maintaining higher ATP content and higher energy levels [7].

Polysaccharides have been widely researched and applied in edible mushroom preservation due to their unique properties and benefits in extending shelf life, maintaining quality, and facilitating operability [3,8,9]. For example, a composite coating of sodium alginate and pholiota nameko peptide has a beneficial effect on maintaining the postharvest storage quality of shiitake mushrooms and can extend their shelf life. Polysaccharides such as chitosan, alginate, pectin, and starch can be formulated into edible coatings, which can be directly applied to the surface of edible mushrooms to achieve preservation [10,11]. These coatings form a protective barrier that reduces moisture loss, minimizes gas exchange, and inhibits microbial growth, thus extending the shelf life of the mushrooms [12]. Additionally, polysaccharide-based materials are widely used in the preparation of edible and environmentally friendly films, as smart and/or general packaging materials in the preservation process of edible mushrooms [13,14]. Some polysaccharides extracted from natural sources, such as β-glucan extracted from the stalks of fresh shiitake mushrooms by our group, have antioxidant and antimicrobial properties and are applied to preserve the freshness of shiitake mushrooms [8,9,15]. These properties inhibit the oxidation of lipids and proteins in mushrooms and control the growth of spoilage-causing microorganisms.

However, the preservation effect and mechanism of action of polysaccharide-based preservation materials have been relatively less explored from the perspective of energy. To gain deeper insights into this critical problem, *Agaricus bisporus* was used to explore and extend the application of edible mushrooms from the energy perspective based on previous studies [8,9,15]. The aim was to assess the efficacy of shiitake stem polysaccharide (LEP)-coated film for the preservation of *Agaricus bisporus* during a storage period of 15 days at 4 °C and to understand the preservation mechanism based on respiration and energy metabolism.

## 2. Materials and Methods

### 2.1. Materials and Chemicals

*A. bisporus* was harvested in January 2024 from a local farm in Xi’an City, Shaanxi Province, China, and transported to the laboratory at a low temperature after harvesting. *A. bisporus* mushrooms of uniform size and without any physical damage were selected and stored in a dark environment at 4 ± 1 °C and 85–90% relative humidity (RH) for 12 h.

SOD enzyme activity, POD enzyme activity, and superoxide anion scavenging rate assay kits were purchased from Nanjing Jiancheng Bioengineering Institute in Nanjing, China. All other chemicals and reagents used were of analytical grade.

### 2.2. Extraction and Isolation of LEP

The isolation of LEP follows a previously reported method by the research group [8,16]. This process involves the conventional method of water extraction, followed by alcohol precipitation. In essence, the shanks of *Lentinula edodes* are first carefully crushed, sieved, and then defatted in ethanol for 48 h. To remove protein content, the Sevag method is employed. Subsequent purification involves using DEAE Cellulose-52 and Sephadex G-100 gel permeation chromatography (Φ50 × 600 mm) to isolate the desired polysaccharide fraction of *Lentinula edodes*, abbreviated as LEP [17].

The polysaccharide content was determined to be 85.34%, signifying its substantial presence within the compound. Furthermore, the molecular weight, a crucial parameter, was determined to be an impressive 6.41 × 10^3^ Da, underscoring the significant size and complexity of LEP. The monosaccharide composition of LEP was thoroughly examined, revealing a ratio of Rhamnose (Rha), Arabinose (Ara), Glucuronic acid (GlcA), Mannose (Man), Fucose (Fuc), Galactose (Gal), and Glucose (Glc) in the composition. The molar ratios were found to be 1.00:1.28:1.79:4.74:6.13:20.62:842.17.

### 2.3. Preparation and Processing of LEP Solutions for Edible Coatings

For the subsequent treatment process, the mushrooms were randomly divided into seven experimental groups. Each group was immersed in LEP solutions at concentrations of 1.0%, 1.5%, or 2.0% (*w/w*) (effective concentration for pre-experimental validation) for precisely 2 min. It is crucial to note that the control group (Con) underwent treatment by means of immersion in distilled water at room temperature. For each treatment group, three independent biological replicates were conducted, and each replicate consisted of ten mushrooms.

### 2.4. Determination of Postharvest Storage Quality Characteristics

#### 2.4.1. Weight Loss

At the beginning of storage and at each subsequent storage interval, a determined quantity of mushrooms from each group underwent careful weighing to determine their weight loss [18]. The calculation of weight loss involved determining the percentage of mass lost relative to the initial weight. The experiment was performed in triplicate.

#### 2.4.2. Textural Characteristics

The assessment of the texture properties of the mushrooms was conducted using the advanced American FTC texture analyzer TMS-PRO (Beijing Yin Sheng Heng Tai Technology Co., Ltd., Beijing, China), ensuring high precision and reliability [19]. The assessment involved using the TPA mode, which simulates the human chewing process. A P/2 probe with a diameter of 2 mm was used to conduct the measurements. The testing procedure followed a well-defined protocol, starting with a pre-test speed of 2 mm/s, followed by a test speed of 2 mm/s, and concluding with a post-test speed of 5 mm/s. The measurement depth was consistently maintained at 10 mm, and the trigger force was set to 5 g.

#### 2.4.3. Soluble Protein Content (SPC)

The quantification of SPC was carried out with precision and adherence to the BCA method [20]. The experiment was conducted in triplicate to enhance the accuracy and reliability of the obtained results. The experiment was performed in triplicate.

#### 2.4.4. Browning Degree (BD)

First, 5 g of the mushroom sample was mixed with chilled distilled water at a ratio of 1:10 (*w/w*). Subsequently, a thorough homogenization process lasting 30 s was performed, followed by centrifugation at 8500× *g* for 15 min. The supernatant obtained from this process was then kept at a constant temperature of 25 °C for 5 min to ensure optimal stabilization. To quantify the degree of browning, absorbance measurements were taken at a wavelength of 410 nm. The browning results are expressed as 10 × A_410_, following standard conventions for reporting such values [8].

### 2.5. Determination of Malondialdehyde (MDA) Content and Electrolyte Leakage Rate

The Nanjing Jiancheng Institute of Bioengineering kit’s instructions were followed to execute the standardized thiobarbituric acid procedure for quantifying MDA content. To assess the electrolyte leakage rate, the research group followed a previously established method [8]. Conductivity measurements were taken by recording the initial value (P0), followed by another measurement (P1) 10 min later. The samples were boiled for 10 min, and the conductivity value (P2) was determined after the temperature dropped to 25 °C. The formula for calculating the relative leakage rate was (P1 − P0)/(P2 − P0), ensuring a standardized approach to assessing electrolyte leakage [21].

### 2.6. Determination of Resistance Indicators

#### 2.6.1. Total Phenolic Content (TPC)

Total phenol content (TPC) was quantified using the well-respected Folin–Ciocalteu method [8]. The experiment was replicated in triplicate to ensure the accuracy and reliability of the results.

#### 2.6.2. H_2_O_2_ Content

To assess H_2_O_2_ content, a mixture of mushrooms (2 g) with 5 mL of 0.1 mol/L phosphate-buffered saline (PBS) at pH 7.0 was prepared for different storage periods. The mixture was ground while surrounded by ice to create a homogenate, then rotated at 1200× *g* for 10 min and separated. The supernatant was used for H_2_O_2_ content analysis [22]. H_2_O_2_ content was quantified using an H_2_O_2_ detection kit (μmol/g) from Shanghai Yuanye Biotechnology Co., Ltd., Shanghai, China.

#### 2.6.3. Peroxidase (POD), Catalase (CAT), Superoxide Dismutase (SOD), Ascorbate Peroxidase (APX), and Phenylalanine Ammonialyase (PAL) Activities

POD activity was evaluated using the guaiacol method. Briefly, mushrooms (2 g) were mixed with 5.0 mL of PBS (pH 6.4) and ground while surrounded by ice to create a homogenate. The mixture was rotated at 4 °C at 12,000× *g* for 10 min and separated to produce the enzyme extract as a supernatant. The reaction proceeded for 2 min, and absorbance at 470 nm was detected and written down every 30 s for a total of 3 min. Units (U), which represent a 0.01 change in absorbance per minute, were used to express the results. CAT and SOD were withdrawn using the approach outlined by Hou et al. [8]. Mushrooms (2 g) were mixed with 5.0 mL of PBS (pH 7.8) and ground while surrounded by ice to create a homogenate. The mixture was rotated at 4 °C 12,000× *g* for 10 min and separated to produce the enzyme extract as a supernatant, used for determining CAT and SOD activities. CAT and SOD activities were determined using CAT and SOD assay kits from Shanghai Yuanye Biotechnology Co., Ltd. These assessments followed the manufacturer’s guidelines, ensuring scientific rigor and precision. The study ensured credibility and robustness of findings related to POD, CAT, and SOD enzyme activities in shiitake mushrooms during different storage intervals. PAL and APX activity was assessed using the method documented by our research group [8]. A change in absorbance of 0.01 U/min at 290 nm was used as the unit of enzyme activity for quantification.

### 2.7. Determination of the Antioxidant Activity

Antioxidant capacity was evaluated following the method of Hu et al. [9] with added clarification of key reaction conditions. LEP was dissolved in distilled water, and assays for DPPH·, ·OH, ABTS^+^·, and superoxide anion were performed under standard postharvest physiology conditions. For example, the DPPH· assay involved reacting the sample with 0.1 mmol/L DPPH ethanol solution in the dark for 30 min at 25 °C, and the ABTS^+^· assay used a working solution adjusted to an absorbance of approximately 0.70 at 734 nm. The hydroxyl radical assay was conducted using a Fenton reaction system, and superoxide anion scavenging was assessed using the pyrogallol autoxidation method. Detailed operational steps are available in the study by Hu et al. [9].

### 2.8. Determination of Respiratory Rate and Enzyme Activities Related to Energy Metabolism

Respiration rate was measured using a fruit and vegetable respiration rate meter (model TZ510325-1, Shandong Fengtou Internet of Things Science and Technology Co., Weifang, China). Two fresh *Agaricus bisporus* mushrooms, totaling 50 g, were analyzed at a flow velocity of 1000 mL/min for 1 min and measured five times. The stable difference in CO_2_ concentration was recorded and used to calculate the respiration rate.

Activities of H^+^-ATPase, Ca^2+^-ATPase, succinate dehydrogenase (SDH), and cytochrome c oxidase (CCO) were measured using a kit from Nanjing Jianjian Bioengineering Institute. A decrease in absorbance of 0.01 was considered one unit of activity. ATP, ADP, and AMP contents were measured using a kit from Jiangsu Enzyme Free Biotechnology Co., Ltd. (Wuxi, China). The energy charge was calculated as follows: *EC* = (ATP + 0.5 ADP)/(ATP + ADP + AMP) [23].

### 2.9. Metabolomics Analysis

The reserved *Agaricus bisporus* samples were flash-frozen in liquid nitrogen, placed in Petri dishes, and freeze-dried in a freeze-dryer for 48 h. Subsequently, the lyophilized material was ground into a fine powder and stored at −80 °C for long-term preservation. A total of 8 mg of *Agaricus bisporus* powder (three groups: 0-day fresh group, 15-day CON group, and LEP group, each with three biological replicates, prepared as described in Section 2.3) was homogenized with 1 mL of extraction solvent (methanol/water, 4:1, *v*/*v*, containing 15 µg/mL tridecanoic acid as an internal standard) in a 1.5 mL microcentrifuge tube. The mixture was subjected to ultrasonication for 2 min (20 kHz, 200 W), followed by precipitation at −20 °C for 10 min. Subsequent ice-water bath-assisted ultrasonication (5 min) and incubation (10 min) were performed. The solution was then centrifuged at 13,000 rpm (15,870× *g*) for 15 min at 4 °C. The supernatant was collected, lyophilized under vacuum, and stored in sealed vials at −80 °C for further analysis. Gas chromatography–mass spectrometry (GC-MS) analysis was conducted using a Shimadzu QCMS-TQ8040NX system (Shimadzu, Kyoto, Japan). Chromatographic separation was achieved on a DB-5MS capillary column (30 m × 250 μm × 0.25 μm) with high-purity helium (99.99%) as the carrier gas in constant flow mode at a linear velocity of 40.2 cm/s and a split ratio of 20:1. The oven temperature program was set as follows: hold at 70 °C for 3 min, ramp to 310 °C at 5 °C/min, and then maintain at 310 °C for 5 min. The injector and transfer line temperatures were set at 300 °C and 250 °C, respectively. For mass spectrometric detection, the electron impact (EI) ion source temperature was 230 °C with an ionization energy of 70 eV, and the full scan mass range was set from *m*/*z* 33 to 600 with a scan cycle of 0.2 s, accompanied by a 3.5 min solvent delay to eliminate solvent peak interference.

### 2.10. Statistical Analysis

The results are presented as the mean ± standard deviation (SD), ensuring a comprehensive and accurate representation of the data. To detect potential differences between the groups, one-way analysis of variance (ANOVA) followed by Dunnett’s multiple comparisons test was used. Visualization and analysis of the data were performed using GraphPad Prism version 9.0 for Windows. Statistical significance was defined as a *p*-value threshold of <0.05. Partial Least Squares Discriminant Analysis (PLS-DA) was performed using SIMCA-P 13.0 software (Umetrics AB, Umeå, Sweden). Potential biomarkers were screened based on two criteria: Variable Importance in Projection (VIP) scores > 1 and *p*-values < 0.05. Heatmaps were generated using the MetaboAnalyst online platform (version 5.0, http://www.MetaboAnalyst.ca).

Compound identification and pathway annotation of candidate biomarkers were further validated against the Human Metabolome Database (HMDB, http://www.HMDB.ca/). Metabolite identification was performed using HMDB due to its broad coverage of small-molecule metabolites commonly detected across plant, fungal, and microbial metabolomics. To improve annotation accuracy, identifications were cross-validated using KEGG and the NIST/Fiehn mass spectral libraries.

## 3. Results

### 3.1. Quality Characteristics of Agaricus bisporus with the LEP Coating

**Figure 1:**
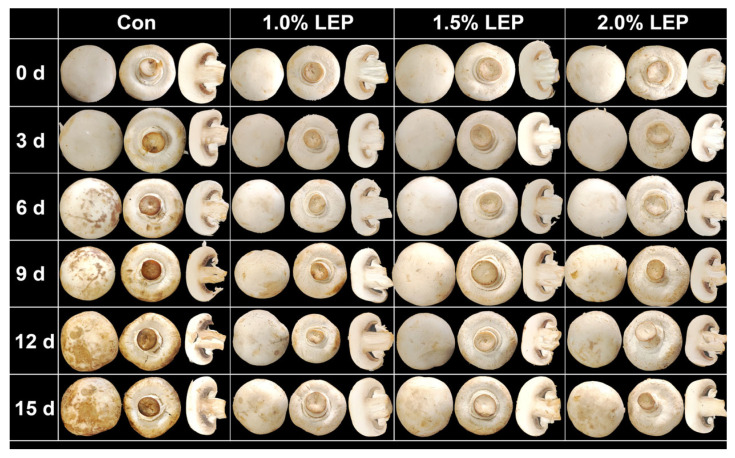
Overall external view of the mushroom during 15 days of storage.

**Figure 2:**
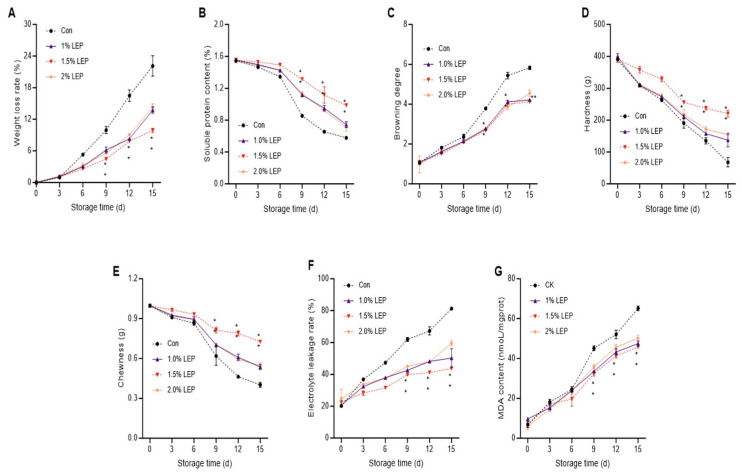
Characterization of the storage quality of *Agaricus bisporus* during 15 days of storage. (**A**) Weight loss rate; (**B**) Soluble protein content; (**C**) Browning degree; (**D**) Hardness; (**E**) Chewiness; (**F**) Electrolyte leakage rate o; (**G**) MDA content illustrate the postharvest quality changes of *Agaricus bisporus* during storage. (Note: (n = 3) Compared with the Con group, ** *p* < 0.01 for the 1.5% LEP group).

Figure 1 illustrates how *Agaricus bisporus* changes in appearance while being stored under different treatments (Con and LEP). As shown in Figure 1, on the 6th day of storage, the mushrooms in the Con group exhibited significant browning and softening, consistent with a decline in commercial quality. In contrast, various concentrations of LEP treatments maintained relatively good color quality throughout the 15-day storage period. As shown in Figure 2A, the weight loss rate directly reflects the quality characteristics of edible mushrooms during storage. By the sixth day of storage, the weight reduction ratio of the Con group hit 5.29% (*p* < 0.01), markedly surpassing the ratio of other treatment groups. After 15 days of storage, the weight reduction ratio of the Con group was as high as 22.12%, rendering it unfit for sale. In contrast, the 1.5% LEP treatment group maintained a relatively low weight loss rate (9.88%, *p* < 0.05), consistent with the preservation process results shown in Figure 1.

As indicated in Figure 2B, the high protein content in *Agaricus bisporus* reflects its nutritional value; therefore, SPC was used to assess the nutritional quality of mushrooms during storage [24]. Throughout the duration of storage, there was a consistent reduction in the SPC levels of mushrooms across all groups treated. On the 15th day of storage, compared to the initial values, the SPC content of the Con group decreased by 62.74% (*p* < 0.01), while that of the 1.0%, 1.5%, and 2.0% LEP treatment groups decreased by 51.99%, 36.36%, and 52.03%, in sequence. As is depicted in Figure 2C, the degree of browning (BD) grew sharply with the extension of the storage period. The browning degree of the control group increased by 83.34%, exceeding levels typically considered visually acceptable in postharvest mushrooms and indicating a marked decline in physical quality, while the BD of the LEP-treated groups rose by 76.67%, 75.01%, and 78.68%, respectively. This result is consistent with the findings shown in Figure 1.

As shown in Figure 2D,E, *A. bisporus* exhibited a consistent decrease in hardness and chewability across all treatment groups throughout the storage period, consistent with conventional expectations [25]. Hardness and chewability are key characteristics for evaluating the texture of *A. bisporus*. Throughout the storage period, the 1.5% LEP treatment group sustained better textural properties, with decreases in hardness and chewability of 43.21% and 26.63%, respectively, whereas the Con group exhibited decreases of 82.73% and 63.59%, respectively (*p* < 0.01).

### 3.2. Characterization of Cell Membrane Permeability

As indicated in Figure 2F, the electrolyte leakage rate of *A. bisporus* increased noticeably (*p* < 0.05, *p* < 0.01) across all treatment groups with extended storage time, indicating that cell membranes were disrupted to some extent in all groups. However, LEP treatments partially alleviated or inhibited the rate of membrane rupture. Notably, post the sixth day of storage, the rate of electrolyte leakage in the Con group markedly exceeded that in the other treatment groups (*p* < 0.01). By the 15th day of storage, the electrolyte leakage rate in the control group reached 81.23%, indicating severe membrane damage and markedly increased leakage of cell contents. In contrast, the electrolyte leakage rates for the 1.0%, 1.5%, and 2.0% LEP treatment groups remained relatively low at 50.23%, 43.71%, and 59.53%, respectively. MDA content, a key indicator of membrane lipid peroxidation, was also used to evaluate changes in cell membrane permeability [26]. As shown in Figure 2G, the MDA content and electrolyte leakage rate displayed a consistent upward trend across the duration of storage. On the 15th day, the MDA content of the Con group was as high as 65.16 nmoL/mgprot (*p* < 0.01), while the MDA content in the LEP-treated groups was 47.48 nmoL/mgprot, 45.78 nmoL/mgprot, and 50.17 nmoL/mgprot, respectively, with no significant differences among them.

### 3.3. Characterization of Antioxidant Activity and Resistance with the LEP Coating

**Figure 3:**
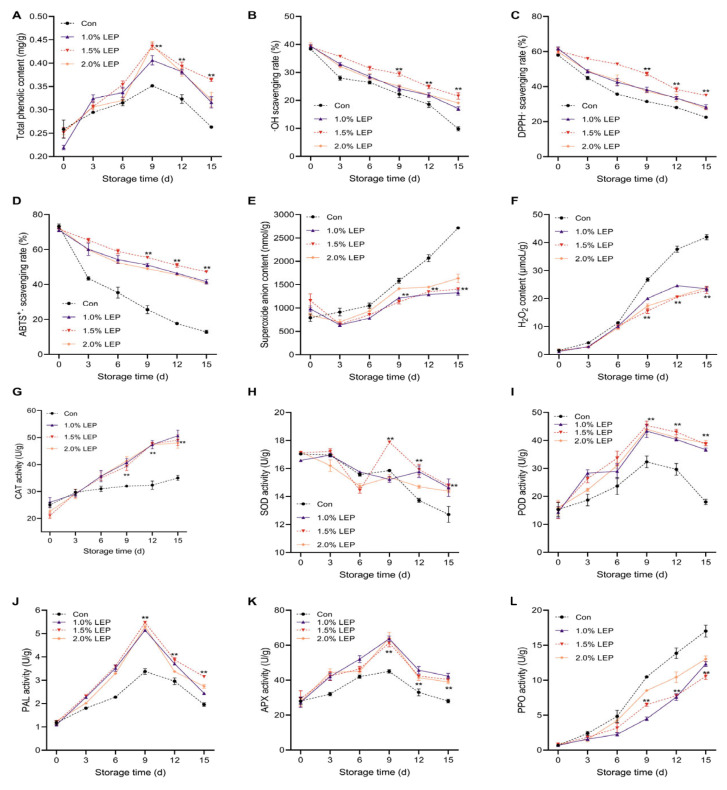
Characterization of enzyme activities related to the antioxidant activity and resilience properties of *Agaricus bisporus* during 15 days storage. (**A**) Total phenol content; (**B**) ·OH scavenging rate; (**C**) DPPH· scavenging rate; (**D**) ABTS^+^· scavenging rate; (**E**) Superoxide anion content; (**F**) H_2_O_2_ content; (**G**) CAT activity; (**H**) SOD activity; (**I**) POD activity; (**J**) PAL activity; (**K**) APX activity; (**L**) PPO activity illustrate the postharvest quality changes of *Agaricus bisporus* during storage. (Note: (n = 3) Compared with the Con group, ** *p* < 0.01 for the 1.5% LEP group).

As shown in Figure 3A, the total phenolic content (TPC) exhibited a significant peak on the ninth day of storage. This peak may be attributed to the role of TPC in antioxidant activity and phenolic metabolism during postharvest senescence.

Notably, the total phenol content in the LEP treatment groups remained high at the storage endpoint, with values of 0.31 mg/g, 0.32 mg/g, and 0.36 mg/g, evidently better than that of the Con group (*p* < 0.01). Figure 3B–E show the changes in ·OH scavenging rate, DPPH· scavenging rate, and ABTS^+^· scavenging rate. All free radical scavenging metrics exhibited a downward trend during the entire duration of storage, except for the superoxide anion content, which initially decreased and then increased. Overall, the 1.5% LEP treatment group demonstrated relatively superior antioxidant activity. On day 15 of storage, the ·OH scavenging, DPPH· scavenging, ABTS^+^· scavenging, and superoxide anion content were 21.65%, 35.11%, 47.23%, and 1408.02 nmol/g, respectively, which were considerably superior to those of the Con group (*p* < 0.01).

CAT acts as a direct scavenger of hydrogen peroxide; thus, both Figure 3F,G were analyzed. As the storage period progressed, hydrogen peroxide levels accumulated within *Agaricus bisporus*, triggering its ROS stress defense system. However, when hydrogen peroxide levels exceeded the mushrooms’ defensive capacity, decay began (Figure 1 and Figure 3F,G). Figure 3F shows that hydrogen peroxide content generally increased during the duration of the storage process. On the 15th day, the hydrogen peroxide content in the Con group was as high as 41.98 μmol/g (*p* < 0.01), whereas in the LEP groups, it was 22.86 μmol/g, 22.73 μmol/g, and 22.81 μmol/g, respectively, with no significant differences among them. Meanwhile, the CAT activity in the Con group was 35 U/g, whereas the LEP treatments maintained relatively high CAT activity.

Figure 3H illustrates that SOD activity generally increased and then decreased across the duration of storage. The 1.5% LEP-treated *Agaricus bisporus* reached peak SOD activity (17.87 U/g) on the ninth day of storage. On the 15th day, the SOD activities for the Con and LEP-treated groups were 12.72 U/g (*p* < 0.01), 14.62 U/g, 14.76 U/g, and 14.38 U/g, respectively, with no significant differences among the LEP-treated groups. Figure 3I–K show that POD, PAL, and APX activities in the process of storage increase before showing an overall decreasing trend, peaking on the ninth day. This trend is consistent with the total phenol content changes (Figure 3A), suggesting a possible connection to the lignification process in *Agaricus bisporus*, though further experiments are needed for confirmation. On the ninth day of storage, the POD activities of the Con and LEP treatment groups were 32.33 U/g (*p* < 0.01), 43.34 U/g, 45.37 U/g, and 44.01 U/g, respectively. PAL activities were 3.37 U/g (*p* < 0.01), 5.14 U/g, 5.47 U/g, and 5.29 U/g, respectively. APX activities were 45.01 U/g (*p* < 0.01), 63.66 U/g, 61.34 U/g, and 64.00 U/g, respectively. In general, *Agaricus bisporus* treated with 1.5% LEP sustained elevated levels of SOD, POD, PAL, and APX activities relative to the Con group, though the LEP concentrations showed no notable variance.

PPO activity is closely associated with browning in *Agaricus bisporus* (Figure 1 and Figure 2C) and reflects changes in key enzymes of the melanin metabolic pathway in edible mushrooms. Figure 3L shows that PPO activity exhibited a continuous increase during the duration of the storage process. From the ninth day forward, the PPO activity levels in the Con group notably exceeded those in the other treatment groups. On the 15th day of storage, PPO activities in the 1.0%, 1.5%, and 2.0% LEP treatment groups were 72.42%, 59.12%, and 74.11% of those in the Con group, respectively.

### 3.4. Changes in the Respiratory Rate and Activity of Enzymes Related to Energy Metabolism

**Figure 4:**
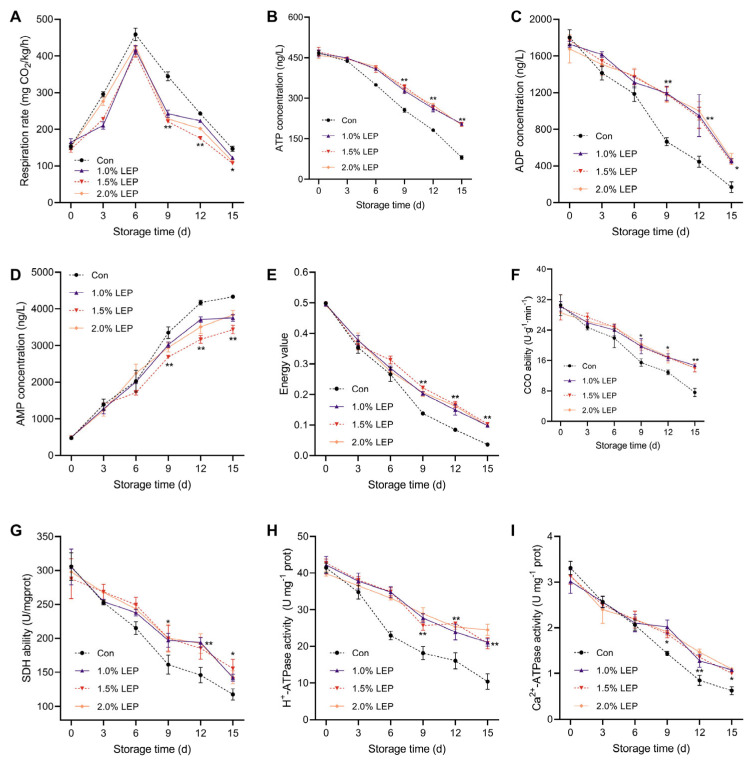
Characterization of the respiration rate, energy substances, and activities of enzymes related to energy metabolism in *Agaricus bisporus* during 15 days of storage. (**A**) Respiration rate; (**B**) ATP concentration; (**C**) ADP concentration; (**D**) AMP concentration; (**E**) Energy value; (**F**) CCO ability; (**G**) SDH ability; (**H**) H^+^–ATPase activity; (**I**) Ca^2+^–ATPase activity illustrate the postharvest quality changes of *Agaricus bisporus* during storage. (Note: (n = 3) Compared with the Con group, * *p* < 0.05 and ** *p* < 0.01 for the 1.5% LEP group).

*Agaricus bisporus*, a typical respiratory leap-type edible mushroom, reached its respiratory peak on the sixth day of postharvest storage (Figure 4A). In contrast, LEP reduced the respiratory peak to appropriate levels. The respiratory peaks on the sixth day were 416.01 mg CO_2_/kg/h, 410.33 mg CO_2_/kg/h, and 436.74 mg CO_2_/kg/h for the Con group (458.66 mg CO_2_/kg/h), and the LEP group, they were 416.01 mg CO_2_/kg/h, 410.33 mg CO_2_/kg/h, and 436.74 mg CO_2_/kg/h, respectively, with no significant difference observed. As shown in Figure 4B–E, the changes in ATP and energy charge significantly decreased throughout the storage period. However, LEP treatment maintained relatively high energy charge and increased the rate of ATP conversion, suggesting that LEP effectively extends the shelf-life of *Agaricus bisporus* through energy regulation. Nonetheless, the selected range of LEP concentration did not achieve a “qualitative breakthrough,” and the LEP components did not significantly differ in their effect on energy within this concentration range. Cytochrome oxidase (CCO), succinate dehydrogenase (SDH), H^+^-ATPase, and Ca^2+^-ATPase play crucial roles as regulatory enzymes in respiratory metabolic processes. As shown in Figure 4F–I, CCO, SDH, H^+^-ATPase, and Ca^2+^-ATPase generally exhibited a decreasing trend with the increasing storage period. On the 15th day of storage, the CCO activity in the LEP treatment group was 1.84, 1.83, and 1.81 times that of the Con group, respectively. The SDH activity was 1.21, 1.32, and 1.22 times that of the Con group, respectively (*p* < 0.05). Similarly, the activity of H^+^-ATPase was 1.21, 1.32, and 1.22 times that of the Con group, respectively (*p* < 0.05). The activity of Ca^2+^-ATPase was 1.21, 1.32, and 1.22 times that of the Con group, respectively (*p* < 0.05).

### 3.5. PLS-DA Analysis

**Figure 5:**
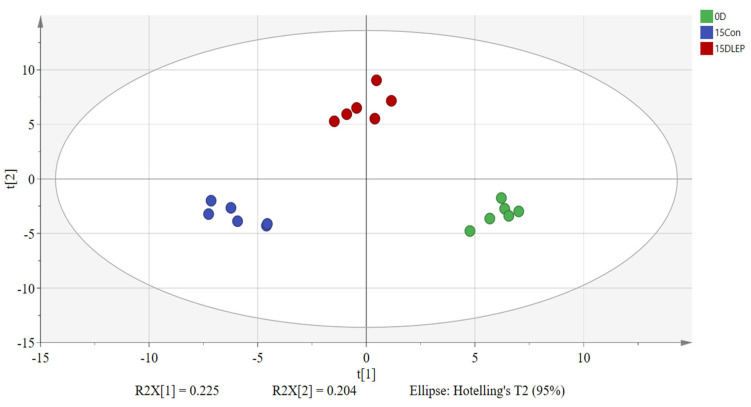
PLS-DA analysis of metabolomics models.

Figure 5 illustrates the PLS-DA score plot of *A. bisporus* under different treatments at day 0 and day 15. The model exhibited strong predictive reliability, with an overall Q^2^ value of 0.923. These results collectively validate the robustness of the PLS-DA model for analyzing metabolomic data in this study.

### 3.6. Differential Metabolite Screening and Metabolic Pathway Analysis

**Figure 6:**
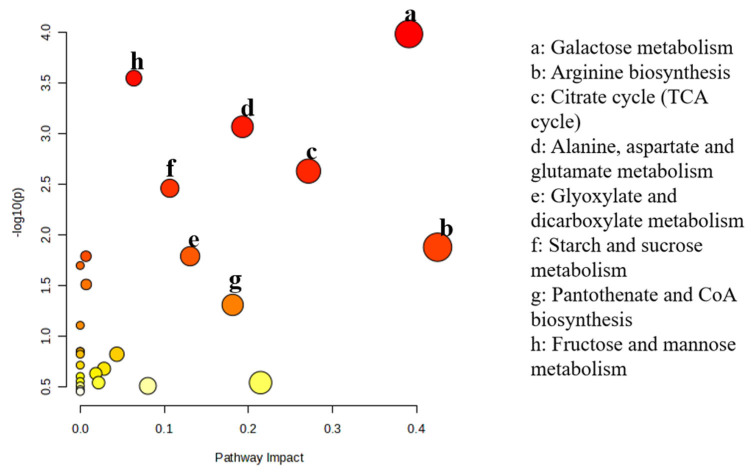
Metabolic pathway analysis.

Based on the *t*-test results, differential metabolites were screened using the criteria of Variable Importance in Projection (VIP) > 1 and *p*-value < 0.05. As summarized in Table 1, a total of 24 significantly altered metabolites were identified between the 15-day Con group and the 15-day LEP group. These differential metabolites primarily included key intermediates involved in energy and amino acid metabolism, such as citric acid, L-serine, malic acid, L-ornithine, and L-isoleucine. As illustrated in Figure 6, the metabolic pathway enrichment analysis of differential metabolites between the 15-day LEP group and the 15-day Con group revealed a total of 28 significantly enriched pathways. Among these, the most highly significant pathways included galactose metabolism, arginine biosynthesis, and the citrate cycle (TCA cycle).

The 1.5% LEP coating synergistically enhances the storage quality of *Agaricus bisporus* through coordinated regulation of three metabolic pathways.

LEP treatment significantly increased the levels of D-galactose and D-glucose (*p* < 0.01), promoting their conversion to UDP-glucose, which reinforces cell wall integrity and structural stability. LEP treatment was associated with increased levels of D-galactose and D-glucose in the galactose metabolism pathway. These changes may indicate enhanced flux through upstream carbohydrate metabolism, although direct conversion to UDP-glucose or effects on cell wall structure were not measured in this study. In parallel, LEP-treated mushrooms exhibited higher CAT and SOD activities. While these findings suggest a possible link between carbohydrate metabolism and antioxidant responses, the current data support correlation rather than a defined causal mechanism.

These changes in α-ketoglutarate, L-ornithine, and L-aspartic acid are consistent with alterations in amino acid and nitrogen-related metabolism, although effects on osmotic regulation or senescence cannot be inferred from the present data.

The LEP coating markedly enhanced the concentrations of citric acid, α-ketoglutarate, and malic acid (*p* < 0.01). Citric acid, as the cycle initiator, accelerates metabolic flux, while α-ketoglutarate bridges amino acid and energy metabolism. Malic acid bolsters antioxidant capacity, collectively sustaining ATP production, energy homeostasis, and suppression of respiratory consumption. The LEP coating orchestrates a synergistic preservation mechanism through systemic regulation of galactose metabolism (cell wall reinforcement/antioxidant defense), arginine biosynthesis (nitrogen metabolism/osmotic balance), and TCA cycle activity (energy homeostasis).

### 3.7. Correlation Analysis of Differential Metabolites with Quality Attributes, Nutritional Properties, and Energy Metabolism-Related Parameters

The integrative analysis indicates that the preservation effect of the 1.5% LEP coating arises from coordinated regulation across energy metabolism, oxidative balance, and membrane integrity. Metabolites associated with the TCA cycle—particularly α-ketoglutarate—showed strong positive associations with citrate, malate, ATP, ADP, energy charge, and the activities of H^+^-ATPase, Ca^2+^-ATPase, SDH, and CCO, suggesting that LEP treatment helps sustain mitochondrial-related energy status during storage. Consistently, respiratory rate exhibited negative correlations with α-ketoglutarate, citrate, malate, soluble proteins, sugars, and phenolic content, implying that LEP is associated with reduced respiratory intensity and, consequently, slower nutrient depletion. Quality deterioration indicators further highlight the biochemical underpinnings of browning and softening. Browning degree was positively related to MDA, H_2_O_2_, weight loss, PPO activity, and electrolyte leakage, while showing negative relationships with antioxidant capacities, soluble protein, firmness, chewiness, L* value, and PAL activity. These patterns illustrate the central involvement of oxidative injury and loss of membrane stability in visual discoloration and textural decline.

**Figure 7:**
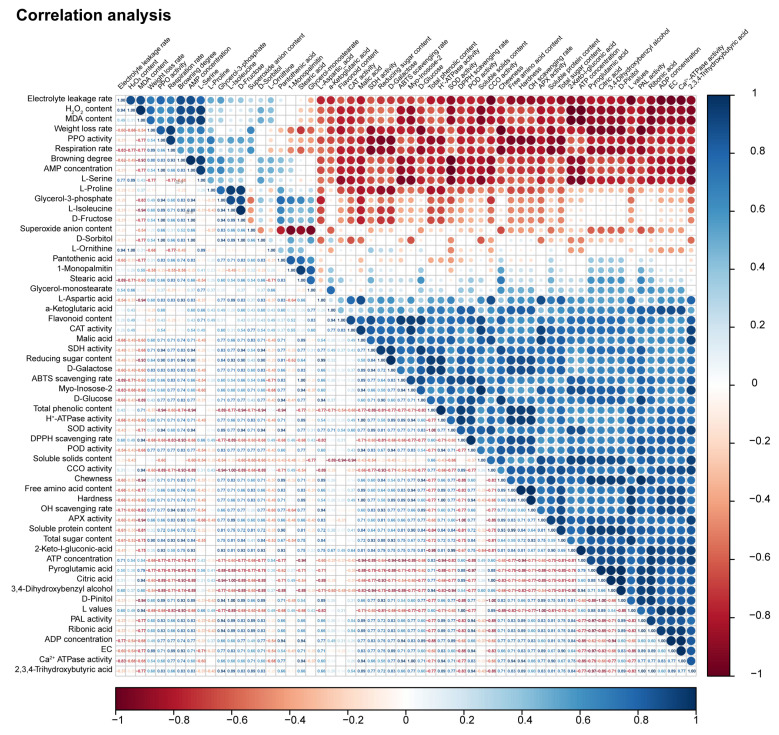
Pearson correlation analysis of physiological biochemical metabolism during storage.

Redox regulation emerged as a key dimension of LEP’s effect. H_2_O_2_ levels were closely associated with markers of oxidative stress and membrane disruption, whereas antioxidant enzymes (CAT, POD, SOD, and APX) and free-radical scavenging activities exhibited strong negative associations with H_2_O_2_ accumulation. The enhanced antioxidant capacity observed in LEP-treated mushrooms therefore reflects a more balanced redox state, helping to suppress the downstream cascade of oxidative damage. Collectively, these findings depict the 1.5% LEP coating as a multi-target intervention that supports energy homeostasis through modulation of TCA-related metabolites, alleviates oxidative and membrane damage that drive quality loss, and strengthens endogenous antioxidant defense pathways. This integrated regulatory network provides a solid theoretical basis for the use of polysaccharide-based coatings in postharvest mushroom preservation (Figure 7).

## 4. Discussion

This research extends and provides mechanistic insights into the group’s previous studies on the preservation of edible mushrooms using shiitake stalk polysaccharides (LEP) [8,9]. Earlier work by the group provided a comprehensive overview of advancements in the use of exogenous polysaccharides to enhance the preservation of edible mushrooms and the development of preservation strategies for shiitake mushrooms [8,9]. Building on the intrinsic capacity of biological tissues to activate stress-response pathways and the role of polysaccharide treatments in enhancing endogenous defense mechanisms, this study provides insights into how LEP may support postharvest quality maintenance [3,9]. This study explores the relationship between polysaccharide intervention and the intrinsic self-defense capacity of mushrooms, mediated through energy metabolism and respiration.

**Figure 8:**
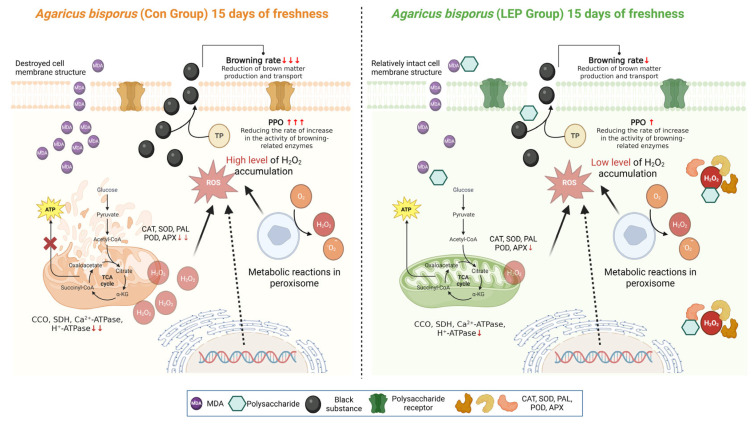
Potential LEP mechanism of action on *Agaricus bisporus* mushrooms mediated through energy metabolism. Created in BioRender. Guo, Y. (2025) https://BioRender.com/d2qiahj (accessed on 15 October 2025).

Key findings from the present study indicate that 1.5% LEP had a significant preservation effect on *Agaricus bisporus*, extending its shelf life by 9 days (up to 15 days). For evaluating the resilience of edible mushrooms in research on plant stress resistance, enzyme measures like POD, CAT, SOD, APX, and PAL play a vital role [3,27,28]. Plants activate a defense regulatory system to mitigate the harmful effects of ROS [6,29,30]. Within this system, SOD is responsible for converting superoxide anion radicals into hydrogen peroxide, while POD and CAT neutralize hydrogen peroxide [23,31]. This pathway has been widely documented, and many studies have investigated ROS metabolism as a strategy for improving freshness [32,33,34]. Accordingly, this paper uses the ROS metabolism process to elucidate the mechanism of LEP in preserving *Agaricus bisporus* (Figure 8).

Another strategy in this paper involves studying energy metabolism to elucidate the mechanism of LEP preservation. It is well known that edible mushrooms require a delicate balance between the energy costs of respiration to thrive [4,29,35]. Thus, respiration must be considered when analyzing mushroom growth. The energy state of edible mushrooms significantly affects their respiration rate; low energy charges promote respiratory metabolism, while high energy charges inhibit it [30,31,36]. The energy charge of a system acts as a marker for its energy condition [37,38]. Experimental data indicate that, compared to the control group, the enhanced energy status conferred by exogenous polysaccharides suppressed respiratory activity on days 9, 12, and 15, thereby reducing metabolic consumption. Chitin- and β-glucan-based cell wall maintenance in mushrooms is energy dependent according to previous studies. While we did not assess cell wall structure directly, the concurrent changes in ATP/ADP levels and texture parameters in our study may indicate an associative relationship, but causality cannot be inferred [39]. Sufficient energy supply, particularly ATP and ADP, is crucial for preserving the structural soundness of cell walls and averting the softening of fruits. The state of energy within cells is controlled by the concentrations of ATP, ADP, and AMP. In our study, decaying *Agaricus bisporus* had lower ATP levels but higher AMP levels. We detected reductions in ATP, ADP, and energy charge during postharvest storage, and LEP treatment mitigated these declines. Therefore, the addition of LEP may have improved this energy metabolism process. Additionally, LEP may affect mitochondrial structure, as inferred from changes in mitochondrial-associated enzyme activities, helping to maintain the integrity of the *Agaricus bisporus* cell membrane and mitochondrial structure, which is essential for preserving the shelf life of edible mushrooms. During respiratory metabolism, *Agaricus bisporus* undergoes nutrient decomposition. The LEP coating modulates the TCA cycle pathway, thereby reducing respiratory rate and effectively suppressing excessive nutrient catabolism. This regulatory mechanism maintains a high-energy state in *Agaricus bisporus* during the postharvest phase, ultimately delaying quality deterioration (Figure 8).

## 5. Conclusions

In this study, 1.5% LEP treatment effectively preserved the storage quality of *Agaricus bisporus* by reducing respiration intensity and enhancing energy-related metabolic activity, thereby extending acceptable freshness up to 15 days. These results suggest that LEP has the potential to improve the nutritional attributes, overall quality, and consumer acceptability of *A. bisporus* during postharvest handling. Consequently, LEP may serve as a promising natural coating material for prolonging the pre-market storage period of fresh mushrooms. However, this study was conducted on a single A. bisporus cultivar obtained from one source and assessed under only one storage temperature (4 °C), which may limit the generalizability of the findings. Moreover, the conclusions regarding mitochondrial involvement are based on biochemical indicators rather than direct structural evidence. Future studies should therefore evaluate LEP efficacy across multiple mushroom varieties, production origins, and storage temperatures and integrate structural or imaging analyses to further clarify the underlying mechanisms. Broader validation will help determine the practical applicability of LEP in commercial postharvest preservation.

## Figures and Tables

**Table 1:** Differential metabolites.

Compound	VIP	*p*-Value
L-Serine	1.795358465	2.13 × 10^−8^
Ribonic acid	1.768454953	4.06 × 10^−7^
3,4-Dihydroxybenzyl alcohol	1.748858063	8.89 × 10^−7^
D-Glucose	1.746762372	1.46 × 10^−6^
2-Keto-l-gluconic acid	1.720553155	3.08 × 10^−6^
D-Galactose	1.718800368	5.44 × 10^−6^
Myo-Inosose-2	1.712736451	1.07 × 10^−5^
Citric acid	1.695682318	2.25 × 10^−5^
D-Pinitol	1.5536861	0.0006228
Pyroglutamic acid	1.541290666	0.00068597
Pantothenic acid	1.516676124	0.0007956
D-Fructose	1.504437432	0.00092833
Glycerol-3-phosphate	1.493856349	0.00094816
L-Isoleucine	1.462528497	0.0018512
L-Ornithine	1.462359356	0.0018526
Malic acid	1.42457479	0.0025271
L-Proline	1.389494792	0.0035705
D-Sorbitol	1.108195734	0.036345
2,3,4-Trihydroxybutyric acid	1.357243758	0.0059284
Glycerol monostearate	1.328366752	0.0089983
L-Aspartic acid	1.323794245	0.010176
Stearic acid	1.300520078	0.01095
α-Ketoglutaric acid	1.240855701	0.012567
1-Monopalmitin	1.223792567	0.015668

## Data Availability

The original contributions presented in this study are included in this article, and further inquiries can be directed to the corresponding authors.

## References

[1] Chen Y., Lin H., Zhang S., Sun J., Lin Y., Wang H., Lin M., Shi J. (2018). Phomopsis longanae Chi-induced disease development and pericarp browning of harvested longan fruit in association with energy metabolism. Front. Microbiol..

[2] Cheng X.Y., Yang S., Fang Q., Dai S.C., Peng X.H., Sun M.Y., Lian Z., Liu Y., Yang J., Xu J. (2023). Biomacromolecule assembly of soy glycinin-potato starch complexes: Focus on structure, function, and applications. Carbohydr. Polym..

[3] Du H.Y., Sun X.L., Chong X.A., Yang M.Y., Zhu Z., Wen Y.Q. (2023). A review on smart active packaging systems for food preservation: Applications and future trends. Trends Food Sci. Technol..

[4] Gong P., Wang X.J., Liu M., Wang M.R., Wang S.Y., Guo Y.X., Chang X., Yang W., Chen X., Chen F. (2022). Hypoglycemic effect of a novel polysaccharide from *Lentinus edodes* on STZ-induced diabetic mice via metabolomics study and Nrf2/HO-1 pathway. Food Funct..

[5] Guo Y.X., Chen X.F., Gong P., Guo J., Deng D., He G.L., Ji C., Wang R., Long H., Wang J. (2022). Effect of shiitake mushrooms polysaccharide and chitosan coating on softening and browning of shiitake mushrooms (*Lentinus edodes*) during postharvest storage. Int. J. Biol. Macromol..

[6] Guo Y.X., Chen X.F., Gong P., Long H., Wang J.T., Deng Z.F., Wang R., Han A., Qi Z., Yao W. (2023). Characterization of an active film prepared with *Lentinus edodes* (shiitake) polysaccharide and its effect on post-harvest quality and storage of shiitake. Int. J. Biol. Macromol..

[7] Guo Y.X., Chen X.F., Gong P., Wang R.T., Qi Z.Y., Deng Z.F., Han A., Long H., Wang J., Yao W. (2023). Advances in Postharvest Storage and Preservation Strategies for *Pleurotus eryngii*. Foods.

[8] Hou F.Y., Yi F.X., Song L.S., Zhan S.Q., Zhang R.F., Han X.B., Sun X., Liu Z.L. (2023). Bacterial community dynamics and metabolic functions prediction in white button mushroom (*Agaricus bisporus*) during storage. Food Res. Int..

[9] Hu S.Q., Hou Y.Y., Zhao L.Y., Zheng Y.H., Jin P. (2023). Exogenous 24-epibrassinolide alleviates chilling injury in peach fruit through modulating PpGATA12-mediated sucrose and energy metabolisms. Food Chem..

[10] Kamali M., Shabanpour B., Pourashouri P., Kordjazi M. (2023). Effect of chitosan-coated Ulva intestinalis sulfated polysaccharide nanoliposome on melanosis and quality of Pacific white shrimp during ice storage. Int. J. Biol. Macromol..

[11] Kong Q., Mu H.L., Han Y.C., Wu W.J., Tong C., Fang X.J., Liu R., Chen H., Gao H. (2021). Biodegradable phase change materials with high latent heat: Preparation and application on *Lentinus edodes* storage. Food Chem..

[12] Li C.-y., Cheng Y., Hou J.-b., Zhu J., Sun L., Ge Y.-h. (2021). Application of methyl jasmonate postharvest maintains the quality of Nanguo pears by regulating mitochondrial energy metabolism. J. Integr. Agric..

[13] Li S.E., Jiang H., Wang Y., Lyu L., Prusky D., Ji Y., Zheng X., Bi Y. (2020). Effect of benzothiadiazole treatment on improving the mitochondrial energy metabolism involved in induced resistance of apple fruit during postharvest storage. Food Chem..

[14] Li Y., Wang F., Xu J.R., Wang T., Zhan J.L., Ma R.R., Tian Y.Q. (2023). Improvement in the optical properties of starch coatings via chemical-physical combination strategy for fruits preservation. Food Hydrocoll..

[15] Lin X.H., Sun D.W. (2019). Research advances in browning of button mushroom (*Agaricus bisporus*): Affecting factors and controlling methods. Trends Food Sci. Technol..

[16] Lin Y.X., Lin H.T., Chen Y.H., Wang H., Lin M.S., Ritenour M.A., Lin Y.F. (2020). The role of ROS-induced change of respiratory metabolism in pulp breakdown development of longan fruit during storage. Food Chem..

[17] Lin Y.X., Lin H.T., Lin M.S., Chen Y.H., Wang H., Fan Z.Q., Ritenour M.A., Lin Y. (2020). Hydrogen peroxide reduced ATPase activity and the levels of ATP, ADP, and energy charge and its association with pulp breakdown occurrence of longan fruit during storage. Food Chem..

[18] Liu Q.Q., Xie H.L., Chen Y.H., Lin M.S., Hung Y.C., Wang H., Fan Z., Lin Y., Lin H.T. (2023). Acidic electrolyzed oxidizing water delayed the breakdown occurrence in pulp of fresh longan by regulating the metabolisms of respiratory and energy. Postharvest Biol. Technol..

[19] Nassarava S.S., Bao N.A., Zhang X.T., Ru Q.M., Luo Z.S. (2024). Evaluation of light irradiation on anthocyanins and energy metabolism of grape (*Vitis vinifera* L.) during storage. Food Chem..

[20] Oktay C., Kahyaoglu L.N., Moradi M. (2023). Food freshness monitoring using poly(vinyl alcohol) and anthocyanins doped zeolitic imidazolate framework-8 multilayer films with bacterial nanocellulose beneath as support. Carbohydr. Polym..

[21] Pei F., Han P., Zhou Z.C., Fang D.L., Mariga A.M., Yang W.J., Ma N., Hu Q.H. (2022). The characteristics of the film assembled by caffeic acid-grafted-chitosan/ polylactic acid and its effect on the postharvest quality of *Agaricus bisporus*. Food Packag. Shelf Life.

[22] Riseh R.S., Vatankhah M., Hassanisaadi M., Kennedy J.F. (2023). Chitosan-based nanocomposites as coatings and packaging materials for the postharvest improvement of agricultural product: A review. Carbohydr. Polym..

[23] Shan Y.X., Li F.J., Lian Q.Q., Xie L.H., Zhu H., Li T.T., Zhang J., Duan X., Jiang Y.M. (2022). Role of apyrase-mediated eATP signal in chilling injury of postharvest banana fruit during storage. Postharvest Biol. Technol..

[24] Shan Y.X., Zhang D.D., Luo Z.S., Li T.T., Qu H.X., Duan X.W., Jiang Y.M. (2022). Advances in chilling injury of postharvest fruit and vegetable: Extracellular ATP aspects. Compr. Rev. Food Sci. Food Saf..

[25] Shan Y.X., Zhang S.T., Li Y., Zhang J., Farag M.A., He J.X., Xiao J., Qu H., Duan X., Jiang Y.M. (2023). The roles of exogenous ATP in postharvest fruit and vegetable: A systematic meta-analysis. Postharvest Biol. Technol..

[26] Shu C., Cao J., Jiang W. (2022). Postharvest vibration-induced apple quality deterioration is associated with the energy dissipation system. Food Chem..

[27] Tan X.-L., Fan Z.-Q., Zeng Z.-X., Shan W., Kuang J.-F., Lu W.-J., Su X.-G., Tao N.-G., Lakshmanan P., Chen J.-Y. (2021). Exogenous melatonin maintains leaf quality of postharvest Chinese flowering cabbage by modulating respiratory metabolism and energy status. Postharvest Biol. Technol..

[28] Wang F., Yang Q., Zhao Q., Zhang X. (2020). Roles of antioxidant capacity and energy metabolism in the maturity-dependent chilling tolerance of postharvest kiwifruit. Postharvest Biol. Technol..

[29] Yang W., Shi C., Hu Q., Wu Y., Fang D., Pei F., Mariga A.M. (2019). Nanocomposite packaging regulate respiration and energy metabolism in Flammulina velutipes. Postharvest Biol. Technol..

[30] Yuan L.B., Liu R.Q., Zhou Y.F., Zhang R.Y., Chen S., Yang Q., Gu Y., Han L., Yan B. (2024). Janus biopolymer nanocomposite coating with excellent antibacterial and water/oxygen barrier performance for fruit preservation. Food Hydrocoll..

[31] Zhang J., Wang C., Chen C., Zhang S., Zhao X., Wu C., Kou X., Xue Z. (2023). Glycine betaine inhibits postharvest softening and quality decline of winter jujube fruit by regulating energy and antioxidant metabolism. Food Chem..

[32] Xia R.R., Hou Z.S., Xu H.R., Li Y.T., Sun Y., Wang Y.F., Zhu J., Wang Z., Pan S., Xin G. (2023). Emerging technologies for preservation and quality evaluation of postharvest edible mushrooms: A review. Crit. Rev. Food Sci. Nutr..

[33] Xue W.H., Zhu J.X., Sun P.D., Yang F.M., Wu H., Li W.X. (2023). Permeability of biodegradable film comprising biopolymers derived from marine origin for food packaging application: A review. Trends Food Sci. Technol..

[34] Yan M., Yuan B., Cheng S.J., Huang H.D., Huang D.C., Chen J.Q., Cao C.J. (2020). Nanocomposite-based packaging affected the taste components of white *Hypsizygus marmoreus* by regulating energy status. Food Chem..

[35] Yang B.Q., Han Y.C., Gao H.Y., Liu R.L., Liu R.H., Xu F., Xiao S., Li B., Chen H.J. (2023). Application of melatonin delays lignification in postharvest water bamboo shoots in association with energy metabolism. Postharvest Biol. Technol..

[36] Zhang W., Zhao H., Jiang H., Xu Y., Cao J., Jiang W. (2020). Multiple 1-MCP treatment more effectively alleviated postharvest nectarine chilling injury than conventional one-time 1-MCP treatment by regulating ROS and energy metabolism. Food Chem..

[37] Zhang Y., Tang H., Lei D., Zhao B., Zhou X., Yao W., Fan J., Lin Y., Chen Q., Wang Y. (2023). Exogenous melatonin maintains postharvest quality in kiwiberry fruit by regulating sugar metabolism during cold storage. LWT.

[38] Zhou Y., Liu X.C., Liang X.Y., Li H.M., Lai J.H., Liao Y.R., Liu K.D. (2024). Biochemical and metabolomics analyses reveal the mechanisms underlying ascorbic acid and chitosan coating mediated energy homeostasis in postharvest papaya fruit. Food Chem..

[39] Zuo C., Hu Q., Su A., Xu H., Li X., Mariga A.M., Yang W. (2021). Nanocomposite packaging delays lignification of Flammulina velutipes by regulating phenylpropanoid pathway and mitochondrial reactive oxygen species metabolisms. Postharvest Biol. Technol..

